# Rheological and Microbiological Characteristics of Hops and Hot Trub Particles Formed during Beer Production

**DOI:** 10.3390/molecules26030681

**Published:** 2021-01-28

**Authors:** Monika Sterczyńska, Marek Zdaniewicz, Katarzyna Wolny-Koładka

**Affiliations:** 1 Faculty of Mechanical Engineering, Department of Food Industry Processes and Facilities, Koszalin University of Technology, 15-17 Raclawicka, PL75620 Koszalin, Poland; 2 Department of Fermentation Technology and Microbiology, University of Agriculture in Krakow, Balicka 122, 30-149 Krakow, Poland; marek.zdaniewicz@urk.edu.pl; 3 Department of Microbiology and Biomonitoring, University of Agriculture in Krakow, Mickiewicza Ave 24/28, 30-059 Krakow, Poland; katarzyna.wolny@urk.edu.pl

**Keywords:** hop pellets, Polish hops, hot trub, rheology, viscosity, waste, beer, microorganisms

## Abstract

During the production of beer, and especially beer wort, the main wastes are spent grain and hot trub, i.e., the so-called “hot break.” Combined with yeast after fermentation, they represent the most valuable wastes. Hot trub is also one of the most valuable by-products. Studies on the chemical composition of these sediments and their rheological properties as waste products will contribute to their effective disposal and even further use as valuable pharmaceutical and cosmetic raw materials. So far, hot trub has been studied for morphology and particle distribution depending on the raw material composition and beer wort extract. However, there are no preliminary studies on the rheological properties of hot trub and hops. In particular, no attention has yet been paid to the dependence of these properties on the hop variety or different protein sources used. The aim of this study was to examine the effect of different hopping methods on hot trub viscosity and beer wort physicochemical parameters. Additionally, the hop solutions were measured at different temperatures. A microbiological analysis of hop sediments was also performed to determine the post-process survival of selected microorganisms in these wastes. For manufacturers of pumps used in the brewing industry, the most convenient material is that of the lowest viscosity. Low viscosity hot trub can be removed at lower velocities, which reduces costs and simplifies washing and transport. The sediments also had similar equilibrium viscosity values at high shear rates.

## 1. Introduction

For many years, industrial waste generation has been increasing at an alarming rate worldwide [1]. In the food industry, including the brewing industry, there are many problems with waste management and disposal. At the same time, they generate significant costs and are an important aspect in brewery operations [2,3]. In particular, this concerns the use of new recipes for beer wort production. Beer consumption is steadily increasing and the brewing industry increases its revenues every year [4,5]. Each brewery attempts to keep waste disposal costs low, and government-imposed legislation has become more stringent over the years [3,5,6,7,8]. The brewing industry consumes significant amounts of water and thus produces large amounts of wastewater—3.3 m^3^ of wastewater per 1 m^3^ of beer [9,10,11]. Furthermore, 51.2 kg of solid waste is produced per 1 m^3^ of finished beer, including hot trub. The biological oxygen demand (BOD) for hot trub is as high as about 110,000 mg-kg^−1^ [7]. CIP (Cleaning in Place) installations or other pipelines transport raw materials, semi-finished products, finished products, and manufacturing waste. The selection of suitable pumps and optimization of their parameters are determined by the medium properties, including those of a rheological nature, e.g., viscosity [12,13,14]. It is also important to review the material before production and use on a larger scale, to design optimal equipment and improve overall process economics [15,16]. In their study, Piepiórka-Stepuk et al. [17] focused on the regeneration of detergents in brewery production at different temperatures. The authors found that the solutions cleaned in the CIP installation in the brewery differ in the degree and type of contamination [17].

During the production of beer, and especially beer wort, the main wastes are spent grain and hot trub, i.e., the so-called “hot break” [3,5,14,18]. Combined with yeasts after fermentation, they represent the most valuable wastes [7]. Hot trub is also one of the most valuable by-products. Studies on the chemical composition of these sediments and their rheological properties as waste products will contribute to their effective disposal and even further use as valuable pharmaceutical and cosmetic raw materials [3,19].

One of the first stages of beer production is the brewing of beer wort. After boiling, the wort is pumped into a whirlpool (the so-called “breaking”), in which the hot trub is separated [20]. Wort clarification in the whirlpool is mainly aimed at reducing the excess protein and tannic substances. Their presence adversely affects a number of processes and the commercial shelf life of the finished product. The hot trub density is higher than the beer wort density and is approx. 1.2–2.25 g-cm^−3^ [21]. Hot trub is a mixture of insoluble, denatured proteins, complex carbohydrates, lipids, tannins, and many other minerals [22,23]. The share of individual components of the sediment varies depending on the raw materials used [24], and is usually as follows: proteins (40 to 70%), bitter substances (7 to 32%), polyphenols (20 to 30%), carbohydrates (4 to 8%), fats (1 to 8%), ash (nearly 5%), and bitter acids and fats (1 to 2%) [23,25]. Wort with high nitrogen content loses about 6% of it due to the precipitation of hot trub during boiling [26]. So far, the sediment has been studied for morphology and particle distribution (by the Shadow Sizing method) depending on the raw material composition and beer wort extract [27]. The hot trub particle size ranges from 30 to 80 µm [23] or up to 200 µm [28]. Studies showed that these particles can be up to 500 µm in size. Most particles are from about 30 to 140 µm [27]. However, the largest estimated diameter values were around 8000 µm [25]. These relationships should be further examined, especially the effect of the hop pellet type on the obtained hot trub viscosity. In addition, the hop solution rheology at different temperatures is an important aspect when using automatic dosing of raw materials nowadays.

For many years now, the rheological properties of raw materials or products have been tested in almost all aspects of the food industry [29,30,31] or food waste [32]. Measurements of wort viscosity provide information on the wort filtration and clarification time in the brewery. The settings of pumps for the transport of wort and waste, e.g., hot trub, also depend on the rheological properties. Many studies have already discussed the phenomenon occurring in the whirlpool and hot trub forming a cone [33,34,35]. However, there are no preliminary studies on the rheological properties of hot trub and hops. In particular, no attention has yet been paid to the dependence of these properties on the hop variety or various raw protein materials (e.g., barley malt) used.

The antimicrobial properties of hops contained in hop sediments and spent hops allow the use of this waste, among others, as a fertilizer or—as noted above—as a pharmaceutical and cosmetic raw material. This method seems to be more reasonable than the application for animal feed purposes, mainly due to the relatively high content of bitter compounds [8]. Fărcaş et al. [36] stated that because of the presence of 2-methyl-3-buten-2-ol, the application of hop sediment as a feed additive is not justified. Hop residue (with its high content of essential oils) may also be successfully used to produce natural, inexpensive, ecological repellents to fight pests in stored food [8,37]. Hop sediments can also be used in medicine as a sedative or in cosmetology, which results from the presence of specific organic compounds [8], as well as its antioxidant and antimicrobial effects [38]. Unfortunately, a large share of hop sediments is still treated as waste and sent to landfills [39]. Therefore, alternative options for the processing of brewing waste are increasingly being proposed, using the antimicrobial properties of hops contained in the sediments. The proposed measures include, inter alia, the possibility of composting hop sediments for later use as a fertilizer in agriculture [40,41]. Another proposed option for the management of hop sediments was studies aimed at evaluating the use of refuse-derived fuels (RDF) and undersize fraction from municipal solid waste (UFMSW) as bulking agents for co-biodrying hot trub. Therefore, the obtained results suggest using RDF as the main component in the process of hot trub co-biodrying [42]. The literature offers many examples of possible ways of managing hop sediments, which are the waste in beer production, showing the real scale of the problem. The in-depth (physico-chemical, rheological, microbiological) characteristics of this raw material is of key importance here, as it enables the creation of effective and economically justified methods of utilization.

The aims of this study are presented below:

(1) It evaluated the influence of different hopping methods on hot trub viscosity and beer wort physicochemical parameters.
(2) The rheological properties of hop solutions were measured at different temperatures.
(3) Microbiological analyses were performed to check whether the appropriate technological (cleanliness of the installation) and hygienic (high temperature) conditions were maintained at all stages of the process, preventing the growth of undesirable microorganisms.

## 2. Material and Methods

### 2.1. Material

The material used in the study was hot trub, i.e., the so-called “break” precipitated while hopping the beer wort made using various recipes. Additionally, recipes for beers from still other hop varieties were prepared and the precipitated sediments were evaluated for selected characteristics and physicochemical properties. Standardized methods according to the EBC (European Brewing Convention) and the most recent research equipment were used for production and physicochemical characterization [43]. Raw materials used in the study included Mep@Pilsner barley malt (malt used for top-fermenting and bottom-fermenting beers, color range: 3.5 to 4.5 EBC units), Mep@Lager (barley malt of 3–3.5 EBCs), Puławski hop variety T-90 (bitter variety from Poland, characterized by fruity-flowery flavor and spicy aroma, alpha-acids of 7.2%), Magnat hop variety T-90 (a super-bitter variety of hops bred in Poland, alpha-acids of 14.0%), Lubelski hop variety T-90 (a super-aromatic variety, alpha-acids of 3.0%), and Fermentis Saflager S-23 beer yeast in dry form for bottom fermentation.

### 2.2. Rheological Properties

The tests were carried out to determine the rheological properties of hot trub and hops dissolved in water at different temperatures (21 °C or 100 °C). A Viscotester iQ rheometer, hot trub container, and water bath were used. Samples of 40 g each were prepared for testing. Before application, the sediment or hops were unified, all lumps were broken, and the separated wort was mixed. Wort sediments from a semi-technical scale were standardized to contain 76% water and 24% dry matter. After placing the sediment in a container, air bubbles were removed. The container was closed and stored in a refrigerator for relaxation. Then, each time after more than 12 h, the test was performed. The Vane-in-a-cup geometry was chosen with a 3 mm gap, as only this geometry allowed measurements. No reagents were used for the rheological tests.

Rheological parameters were determined by the hysteresis loop test. This test allows the viscosity changes to be determined and the thixotropy to be evaluated. Thixotropy means that under the conditions of isothermal flow of the liquid previously at rest for a longer time at a constant shear rate, the tangential stress decreases reversibly with time. Thixotropy is defined as any process in which, due to the destruction of the internal structure of a system, the internal friction of a liquid decreases isothermally with the passage of shear time, as well as a time-measurable slow return to its original consistency at rest.

Measurements of hop solution were carried out at three temperatures: 15 °C, 60 °C, and 80 °C. On the other hand, measurements of the hot trub viscosity were carried out at 20, 40, 60, and 80 °C. Both tests were conducted under controlled rate of rotation (CR) conditions, with the following settings: increasing shear rate—$\dot{ɣ}$ 0 1/s—50 1/s linearly for a time of 100.00 s and decreasing shear rate—$\dot{ɣ}$ 50 1/s—0 1/s linearly for a time of 100.00 s.

Rheological measurements were taken with a Thermo Scientific HAAKE Viscotester iQ rotational rheometer with a Peltier system for temperature control. The sediment was examined in a vane cup configuration. The sediment was heated in a water bath to the desired temperature. Wort and mash were studied in a co-axial cylinder system with a double gap. Measurements of wort and mash viscosity were carried out at a shear rate of $\dot{ɣ}$ 1000 1/s for a temperature changing linearly from 0 °C to 80 °C with 0.2 °C/s for 300 s.

### 2.3. Dry Matter Content

In order to determine the dry matter content, it was necessary to dry the hot trub. An electronic scale, a scaling container, and a moisture analyzer were used. A total of 3 g of samples of hot trub, hops, and yeast were taken and transferred to the scaling container, which was then placed in the moisture analyzer. After drying, the percentage of moisture was recorded and the dry matter content was calculated.

### 2.4. Total Protein Content

Protein was determined by the Kjeldahl method, which was carried out in 3 stages, including sample mineralization, distillation, and titration. This test involved the determination of nitrogen compounds in the protein. Reagents used in the test included 4% boric acid (H_3_BO_3_), concentrated sulfuric acid, 0.1 M hydrochloric acid (HCl), 33% sodium hydroxide (NaOH), a catalyst, and distilled water. A 3.0 g sample of hot trub was weighed on the electronic scale and transferred to the distillation flask. A catalyst and 10 mL of sulfuric acid were then added and placed in the digestion flask. After mineralization, the sample (dark brown color) was allowed to cool. After cooling, 20 mL of distilled water with phenolphthalein was added to the distillation flask and placed in the distillation apparatus. Boric acid (40 mL) and 4 drops of Tashiro indicator were measured into the 250 mL flask. After distillation, the 0.1 M HCl sample was titrated until the color changed.

On the basis of the titration results obtained, the amount of nitrogen in the tested material was calculated as follows:

d = ((a − b)∙n∙14)/(1000∙m)∙100 (1)

where

a—volume of solution used for titration;
b—volume of solution used for blank test;
n—molarity;
m—weight of sample; and
14—amount of nitrogen, constant.

The nitrogen results obtained were converted to protein by multiplying them by a conversion factor of 6.25 for all results.

### 2.5. Extract

Extract of the obtained cold wort (beer wort) was analyzed with a Hanna Instruments refractometer of the HI 96801 type. The measurement was read in Plato degrees. Deionized water was used for calibration.

### 2.6. Microbiological Analysis of Hot Trub

The samples of hot trub were collected in sterile containers on the same dates as the material for other analyses. The samples were subjected to microbiological analysis using the serial dilution method by Koch. Microbiological analyzes, performed in triplicate, included the assessment of the number of selected groups of microorganisms, i.e., vegetative and endospore bacteria (TSA, BTL Poland, grown at 37 °C for 24 h), mold fungi (MEA, BTL Poland, grown at 24 °C for 5 days), and actinomycetes (Pochon’s agar, BTL Poland, grown at 28 °C for 7 days). The number of vegetative bacteria and spores testifies to the abundance of nutrients easily available for the microorganisms in raw materials. A plurality of bacteria, fungi, and actinomycetes also indicates favorable conditions (temperature, substrate pH, humidity) for the growth and development of microorganisms. Potentially pathogenic bacteria were also determined: *Staphylococcus* spp. (MSA agar, BTL Poland, grown at 37 °C for 24 h), *Escherichia coli* (TBX agar, BTL Poland, grown at 44 °C for 24 h), *Salmonella* spp. and *Shigella* spp. (SS agar, BTL Poland, grown at 37 °C for 24 h), *Enterococcus faecalis* (SB agar, BTL Poland, grown at 37 °C for 48 h), *Pseudomonas aeruginosa* (CN agar, BTL Poland, grown at 37 °C for 48 h), *Proteus spp.* (Nogrady agar, BTL Poland, grown at 37 °C for 48 h), and *Clostridium perfringens* (SC agar, BTL Poland, grown at 37 °C for 24 h), whose presence may pose a threat from an epidemiological point of view and is an important signal informing about microbial contamination [44]. After the incubation period, the grown colonies were counted and the results reported in colony forming units per gram of dry matter of the sample (CFU·g^–1^ d.m.).

### 2.7. Statistical Analysis

Each variant of the experiment was carried out in 5 repetitions. The obtained results were grouped and their mean values and standard deviation were determined. The obtained values were compared and subjected to statistical interpretation. The significance of the effect of the examined variables on protein, dry matter, and extract of beer worts, hops, and hot trub was determined by single-factor and two-factor ANOVA analysis. The significance of differences between means was verified by Duncan’s test (*p* < 0.05). Statistical analysis was performed using the Statistica 13 software from StatSoft.

### 2.8. Experiment Description

Beer wort and hot trub precipitated from it were produced in the laboratory with the use of a Speidel–Breumeister mashing and brewing boiler on a semi-technical scale. Clarified wort and a sample after secondary fermentation was obtained from the industrial brewery. After preliminary tests, the brewing process resulted in the production of 100% Mep@Pilsner malt and 100% Mep@Lager malt with Puławski hops, which was characterized by the highest amount of protein and dry matter.

In order to obtain optimal conditions for enzymes, the mashing of each wort was carried out with guidelines similar to those for the production of congress wort.

The temperature range included 45–50 °C—for proteolytic enzymes and β-glucanases, 62–65 °C—the optimal temperature for β-amylase activity, 70–75 °C—the optimal temperature for α-amylase activity, and 78 °C—the temperature of the mashing end (mash-off) and mash lautering. Each time, iodine tests were carried out in order to check the quality of the mashing process (mashing saccharification). Determining the saccharification rate is very important. An iodine test is the most commonly used method for determining the saccharification of mash. The mash is considered to be saccharged when there is no longer any change in its color after the addition of iodine solution. Ten min after beginning the mash a drop of mash is transferred to the porcelain spot plate and a drop of iodine solution is added. The test is repeated at 5 min intervals until saccharification is complete and a clear yellow area is obtained. The result is reported as “less than 10 min,” “10 to 15 min,” etc. If saccharification is incomplete after one hour this must be stated.

The raw material input of individual variants of worts from which precipitated hot trub was examined was 6 kg of ground barley malt (Mep@Pilsner malt or Mep@Lager), 60 g of hops, and 33 L of water (of which 5 L were used for sweetening).

The experimental variants were different boiling times (60 or 30 min), wet hopping, and the addition of hops (dry hopping) to the brewery wort (made from 100% Mep@Pilsner malt) sample after secondary fermentation.

Adding hops after boiling and cooling the wort was intended to increase the level of hoppy aroma in the beer without increasing the bitterness. This treatment is more often used in the production of top-fermented beers. A slightly different late hopping technique is used for bottom-fermenting beers. Very often hops are used during secondary fermentation or lagering.

Additionally, an experiment was carried out to evaluate the reaction of the hop pellets to the dissolution of the material in water at different temperatures (21 °C and above 100 °C).

The sample identification system used is presented in Table 1.

## 3. Results and Discussion

The following subsections present and discuss the results of the conducted analyses of hot trub in different experimental variants. The study does not include a section on the results of microbiological analyses, because no microbiological contaminants were determined in the studied hop sediments, which proves their full sterility and lack of microbial survival. Due to its properties (low pH, alcohol content, anaerobic conditions, and aseptic effect of the bitter substances contained in the hops, as well as the low content of nutrients consumed by the yeast in fermentation), beer largely defends itself against the development of microbial infections [45,46]. Moreover, in most cases, the activity of microorganisms in the brewing industry is desirable (beer is the result of the fermentation conducted by them). On the other hand, harmful microflora can also be encountered in beer, which can not only survive, but also multiply in it and release its metabolic by-products into beer, thus causing beer spoilage, which manifests itself in sensory changes (unwanted changes in taste and smell) [45,47]. That is why this study assessed the occurrence of selected microorganisms with the potential to form infections and survive in unfavorable environmental conditions thanks to, inter alia, the ability to create spores, shells, and chlamidospores.

### 3.1. Dry Matter

**Figure 1:**
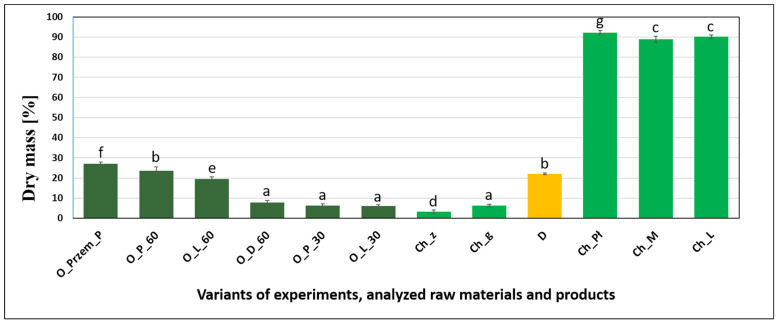
Dry matter of precipitated hot trub, yeast, and raw materials (n = 5, α = 0.05; homogeneous groups of results within a given parameter are marked with letters).

Figure 1 shows the dry matter content in precipitated sediments and selected raw materials. The lowest percentage of dry matter was determined in hop pellets immersed in water at 21 °C (3.37%) compared to hops (Ch_g) with water above 100 °C (6.31%). The dry matter values were similar for variants with hop pellets Ch_g, with precipitated sediments after 30 min of boiling, and after dry hopping. These values ranged from 6 to 7.7%. The other sediment variants had a dry matter content twice as high. For sediments after 60 min of boiling, the highest value (23.52%) of the examined parameter was obtained in the variant with Puławski hops boiled from wort with Pilsner malt; this value was similar to that obtained for beer yeast. The highest dry matter contents were recorded in hop pellets. Among them, the variant obtained from Puławski hops had the lowest moisture content (d.m. 92.32%). The results were similar for the other two variants.

### 3.2. Protein

Hot trub separation can be achieved by sedimentation, centrifugation, or filtration of the beer wort. The most effective precipitation is for hopped wort. Malting barley contains as much as 92% of nitrogen compounds in the form of proteins, mainly glutelins, i.e., simple proteins. They do not pass into the solution because they remain almost whole in the spent grain. Other protein compounds are prolamines (insoluble in water, soluble in alcohol), largely remaining in the spent grain, and globulins, also called “edestins,” (soluble in diluted salt solutions and in mash, heat coagulating, do not precipitate completely). The smallest proportion of barley is made up of albumin (only 11% of proteins), which precipitates completely when boiled. It is worth noting that during malting and mashing, the content decreases. During wort boiling, when the sediment is precipitated, the hop oil components are dissolved and transformed. They are responsible for the formation of the characteristic hoppy smell and taste in beer. These transformations depend on the wort chemical composition and pH [20,48].

**Figure 2:**
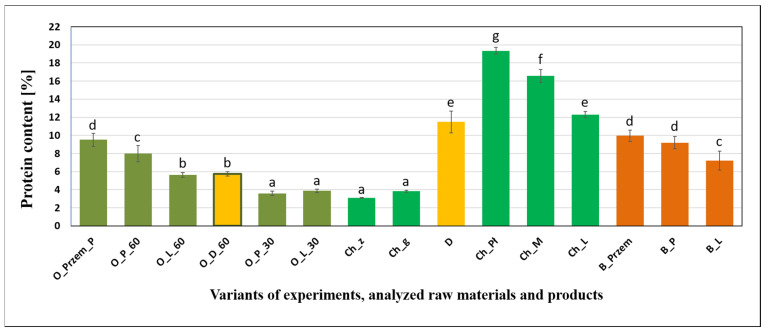
Protein content in beer wort, hot trub, yeast, and raw materials (n = 5, α = 0.05; homogeneous groups of results within a given parameter are marked with letters).

Figure 2 shows a chart of the total content of protein from precipitated sediments and selected raw materials.

The lowest total protein content was determined for hop pellets immersed in water at different temperatures and in sediments precipitated during 30 min of boiling, regardless of beer wort variants. These values oscillated between 3.02 and 3.82%. Among hot trub, the highest protein content was observed for industrial sediment. In the case of variants produced in the laboratory, the highest protein content was found in the sediment with Pilsner malt precipitated after 60 min of boiling (7.98%). The protein values in beer worts and in the sediments were similar depending on the experimental variant. In the case of hot trub after 60 min of boiling, the highest parameter value was obtained in the variant with Puławski hops (23.52%) and it was similar to the mass obtained from beer yeast. The highest protein values were recorded in hop pellets. Among them, the variant with Puławski hops had the highest protein content (19.34%). On the other hand, among the hop pellets in general, the lowest protein content was recorded in Lubelski hops (12.31%).

### 3.3. Extract Content

**Figure 3:**
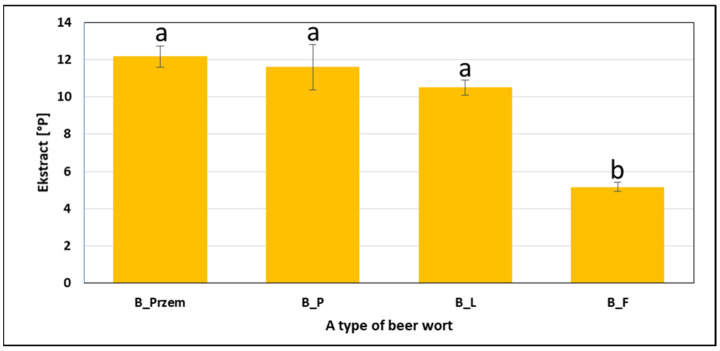
Beer wort extract (n = 5, α = 0.05; homogeneous groups of results within a given parameter are indicated with letters).

The beer wort extract concentration is one of the most important quality parameters and can indicate the brewery’s efficiency [49,50]. It is directly related to the amount of fermenting sugars and is therefore very important for the fermentation process [49]. Figure 3 shows a chart of the extract of worts produced with 100% Pilsner malt and Puławski hops after 60 min of boiling.

There were no statistically significant differences in the results obtained for beer wort extract produced with 100% Pilsner malt and Puławski hops after 60 min of boiling, as it ranged from 10.71 to 12.5 °P. On the other hand, a much lower value of the examined parameter was obtained for wort after dry hopping (5.2 °P). Many authors argued that the distribution of hot trub particles depended on the extract [27], whereas it did not have a major impact on the rheological properties. This variable did not introduce additional differentiation and was the same for all variants without yeast.

### 3.4. Viscosity and Thixotropy

**Figure 4:**
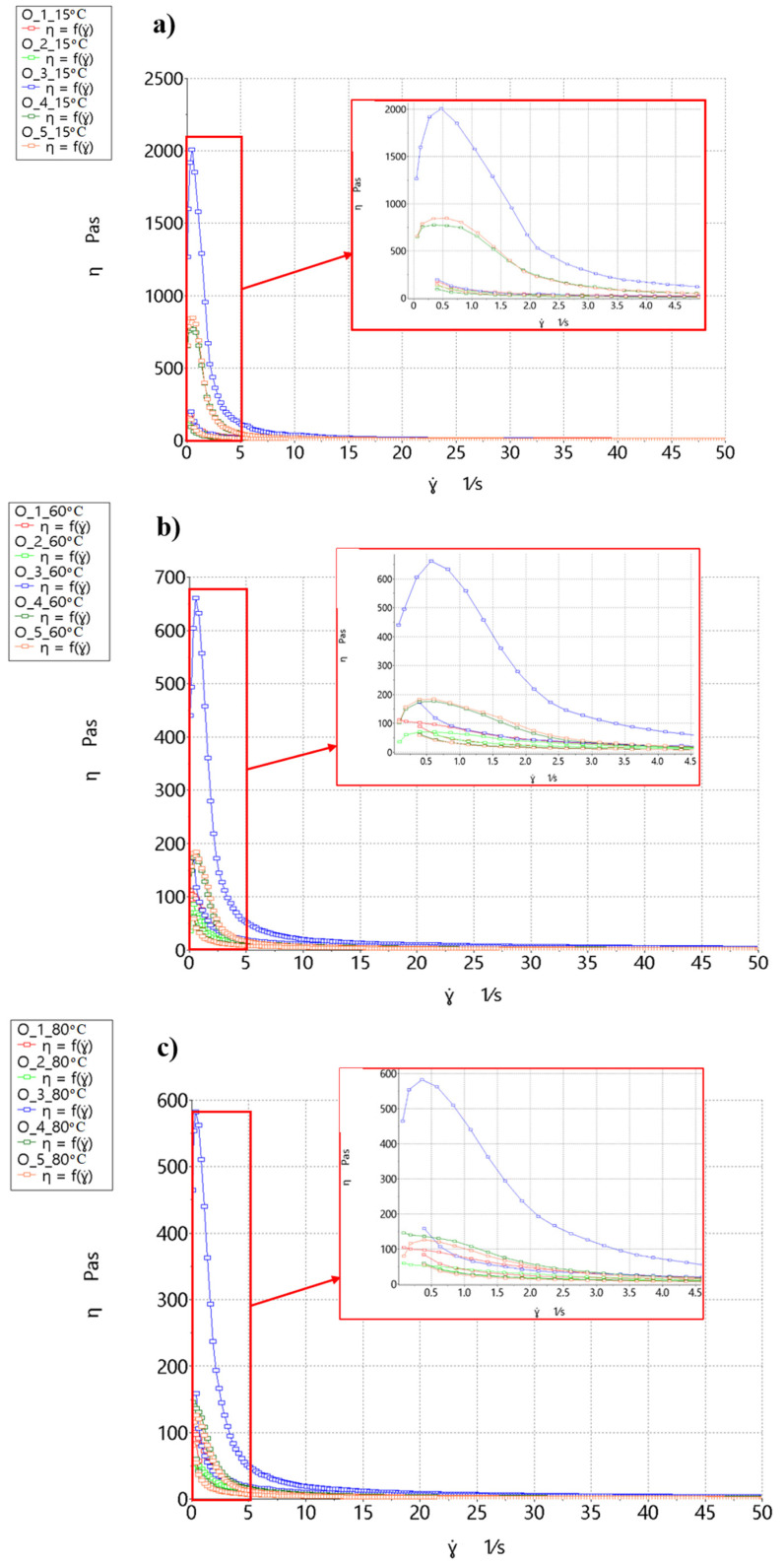
Viscosity in the form of a thixotropy loop of hot trub depending on the temperature: (**a**) 15 °C, (**b**) 60 °C, and (**c**) 80 °C.

The measurements made with the rheometer determine the relationship between the values of tangent stress τ and shear rate $\dot{ɣ}$. The flow curve shows the relation between stresses as a function of τ = f($\dot{ɣ}$). For a Newtonian fluid, this is a straight line passing through the beginning of the coordinate system. Any other curve describes a non-Newtonian fluid [51,52]. A viscosity curve can also be plotted, which represents the dependence of viscosity η on shear rate as a function of η = f($\dot{ɣ}$). The two curves are equivalent. In rheometric measurements, a flow curve is first obtained, which can be converted to a viscosity curve. A chart of the viscosity changes in the form of a hysteresis loop for hot trub from different wort variants is shown in Figure 4.

Hot trub is a non-Newtonian fluid with two characteristic points—maximum viscosity and equilibrium viscosity. Maximum viscosity can be related to yield point: As the maximum value is exceeded, hot trub begins to flow. In turn, equilibrium viscosity is reached as shear rate tends to infinity. The highest maximum viscosity (η_max_) value at all temperatures was obtained in the sediment precipitated from Pilsner malt wort, mixed with yeast, and boiled for 60 min (**O_3**). At 15 °C, the η_max_ was 2008.3 Pa·s; at 60 °C, the value decreased to 660.9 Pa·s; and at 80 °C, it was 464.9 Pa·s.

The lowest maximum viscosity characterized hot trub precipitated from Pilsner malt worts and boiled for 60 min (**O_2**). For this sediment, the maximum viscosity value at 15 °C was 588.1 Pa·s; at 60 °C, η_max_ decreased to 70.7 Pa·s; and at 80 °C, η_max_ was 59.5 Pa·s.

The sediment precipitated from Lager malt wort boiled for 30 min (**O_5**) had the second highest maximum viscosity value. For this hot trub at 15 °C, the maximum viscosity value was 847.2 Pa·s; at 60 °C, η_max_ decreased to 183.9 Pa·s. At 80 °C, η_max_ decreased to 126.4 Pa·s.

The sediment from Pilsner malt wort boiled for 30 min had the third highest value of η_ma_x. The exception was the temperature of 80 °C, at which the **O_4** sample had a higher viscosity than the **O_5** sample. For the **O_4** sediment at 15 °C, the maximum viscosity was 773.7 Pa·s; at 60 °C, η_max_ decreased to 176.4 Pa·s. At 80 °C, η_max_ decreased to 145.2 Pa·s. The **O_1** sediment was precipitated from Pilsner malt worts and boiled for 60 min. For this hot trub at 15 °C, the maximum viscosity was 616.3 Pa·s; at 60 °C, η_max_ was 112.2 Pa·s. At 80 °C, η_max_ decreased to 104.7 Pa·s. In all analyzed sediment variants, the η_max_ value decreased as the temperature increased. The highest temperature drop was observed for the **O_2** hot trub, whose viscosity decreased by 90%. On the other hand, the **O_3** hot trub viscosity decreased by 76%. In other sediments, η_max_ decreased at 80 °C compared to 15 °C by nearly 82%. The addition of yeast to hot trub noticeably thickened the sample. Boiling for 30 min gave hot trub a higher maximum viscosity than boiling for 60 min. A similar effect was observed for the sediment precipitated form Pilsner malt compared to Lager malt.

As the shear rate increased, the sediments became thinner. At the shear rate of $\dot{ɣ}$ = 50 s^−1^, the sediment reached the so-called “equilibrium viscosity” η_eq_. The order of and the tendency for value changes with the increase in temperature were the same as for maximum viscosity. Here, also at 80 °C, the **O_4** sediment had higher viscosity than **O_5**, in contrast to 15 and 60 °C.

The η_eq_ values at

- 15 °C were 4.70 Pa·s (**O_5**), 3.38 Pa·s (**O_4**), 3.25 Pa·s (**O_1**), 2.59 Pa·s (**O_3**), and 2.26 Pa·s (**O_2**);
- 60 °C were 4.26 Pa·s (**O_5**), 3.04 Pa·s (**O_4**), 2.82 Pa·s (**O_1**), 2.15 Pa·s (**O_2**), and 2.05 Pa·s (**O_3**); and
- 80 °C were 3.83 Pa·s (**O_3**), 1.49 Pa·s (**O_5**), 1.75 Pa·s (**O_4**), 1.23 Pa·s (**O_1**), and 1.19 Pa·s (**O_2**).

A comparison of the η_max_ and η_eq_ values shows that the wort boiling time and the malt type affected the maximum viscosity value. With shorter boiling times, the Lager malt trub had a higher viscosity, whereas with longer boiling times, the opposite applied. Mixing the sediment with yeast resulted in a more than three-fold increase in the maximum viscosity at 15 °C, an almost six-fold increase at 60 °C, and a four-fold increase at 80 °C. No significant increase was noted for equilibrium viscosity.

The sediments had substantial thixotropy, which made them sensitive to the deformation time. From the hysteresis test, the ∆A parameter was obtained, i.e., the area between the rising and falling curve. The tendency for changes in the ∆A value was the same as for the viscosity value. The highest values were obtained at 15 °C and the lowest at 80 °C. The highest hysteresis loop area values were observed for the **O_3** sediment: At 15 °C, ∆A was 9171 Pa/s; at 60 °C, it was 4242 Pa/s; and at 80 °C, the parameter value was 3287 Pa/s. For **O_5**, ∆A was 3721 Pa/s at 15 °C, 1076 Pa/s at 60 °C, and 729.3 Pa/s at 80 °C. For the **O_4** sediment, ∆A was 3521 Pa/s at 15 °C, 1016 Pa/s at 60 °C, and 789.3 Pa/s at 80 °C. The penultimate was the **O_2** sediment, for which ∆A was 879.5 Pa/s at 15 °C, 534.6 Pa/s at 60 °C, and 562.2 Pa/s at 80 °C. The lowest hysteresis area value was observed for the **O_1** sediment, for which ∆A was 105.6 Pa/s at 15 °C, 433.4 Pa/s at 60 °C, and 239.5 Pa/s at 80 °C.

Thixotropy, like viscosity, depended on the boiling time and the malt type. The sediment was also characterized by an initial increase in viscosity at low shear rates. The addition of yeast caused a significant increase in the hysteresis loop surface area by increasing the maximum viscosity value. The return curves of all hot trubs, except **O_3**, were similar.

**Figure 5:**
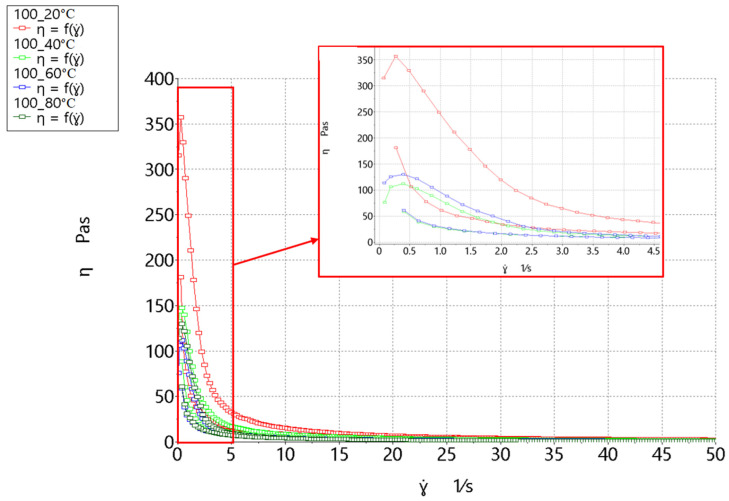
Viscosity and thixotropy of hot trub precipitated from 100% Pilsner malt boiled for 60 min and collected from the industrial brewery at 15 °C, 40 °C, 60 °C, and 80 °C.

Figure 5 shows charts of viscosity changes in the form of a hysteresis loop area of the sediment precipitated from 100% Pilsner malt boiled for 60 min and from wort collected from an industrial brewery.

For industrial sediment, the highest maximum viscosity value was obtained at 15 °C (357.3 Pa·s) and the lowest at 60 °C (111.9 Pa·s.) After heating to 80 °C, η_max_ of 129.8 Pa·s was observed. Such an increase in viscosity can be explained by the swelling of sediment conglomerates; however, no such behavior was observed in laboratory sediments. Their maximum viscosity value was significantly lower than that of semi-technical laboratory sediments.

Industrial sediment had the highest thixotropy value at 15 °C (ΔA = 2028 Pa/s). On the other hand, the lowest value (ΔA = 63.69 Pa/s) was recorded at 60 °C. When heated to 80 °C, the hysteresis loop area value was 260.6 Pa/s. The equilibrium viscosity values decreased with increasing temperature: at 15 °C, η_eq_ = 2.86 Pa·s; at 60 °C, η_eq_ = 1.37 Pa·s; and at 80 °C, η_eq_ = 1.16 Pa·s.

**Figure 6:**
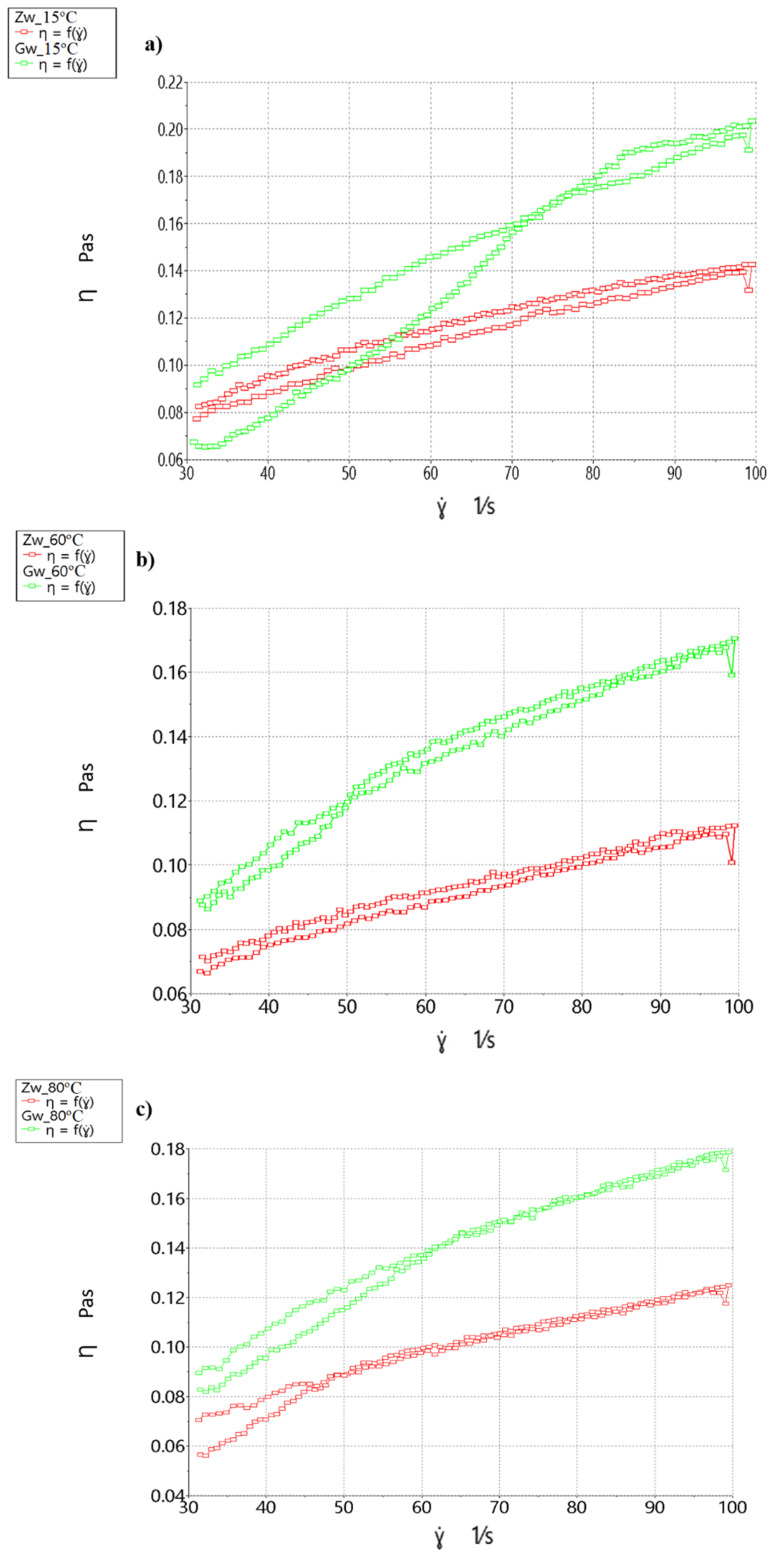
Viscosity and thixotropy loop of hops immersed in water, depending on the experimental variant, at (**a**) 15 °C, (**b**) 60 °C, and (**c**) 80 °C.

Figure 6 shows charts of viscosity changes in the form of hysteresis loops from hops immersed in water at different temperatures.

The highest viscosity at all temperatures was obtained for hops immersed in water at 100 °C (Gw). This was due to the greater swelling of hop particles. The increase in the suspension viscosity with increasing shear rate resulted from mixing. It was therefore not a typical shear thickening. Hence, the decrease in viscosity on the return curve was a result of the hop particle sedimentation. The viscosity value remained at a similar level (0.09 Pa·s) regardless of the material heating temperature.

When tested at 15 °C, the highest thixotropy (ΔA = 28.43 Pa/s) was discovered for hops immersed in water at 21 °C (Zw). The lowest thixotropy (ΔA = 14.08 Pa/s) was found in hops immersed in water at 100 °C (Gw).

The tests at 60 °C and at 15 °C revealed the highest thixotropy (ΔA = 16.62 Pa/s) in hops immersed in water at 21 °C (Zw). The lowest thixotropy (ΔA = 13.13 Pa/s) was found in hops immersed in water at 100 °C (Gw).

Hops can be added via pumps, and thus the viscosity is important. When tested at 80 °C, the highest thixotropy (ΔA = 5.94 Pa/s) was found in hops immersed in water at 21 °C (Zw). The lowest thixotropy (ΔA = 0.1761 Pa/s) was found in hops immersed in water at 100 °C (Gw). Small hysteresis areas suggest that there was no destruction of the hop particle structure. The suspension created by immersion in cold water was more stable. The least stable suspension was obtained at 15 °C for hops immersed in hot water.

Sewage sludges are non-Newtonian fluids [53]. Studies suggested that hot trub could also be composted and processed into fertilizer or used as a source of bioactive compounds [54]. Rheology is used to assess the quality of sewage sludge. Other authors studied the rheology of mixed primary and secondary miscellaneous pre-industrial sludge and how it depends on the solid content and temperature [55]. The rheological properties depend on the structure and surface characteristics of aggregates [56,57].

Cao et al. (2016) studied sewage sludge for 4, 7, 8, 9, and 10% total solids at 20 °C, 35 °C, and 55 °C. The apparent viscosity for these variants decreased noticeably at the beginning of shearing, and then tended to a relatively constant value. A similar tendency was noted for hot trub. Viscosity values ranged from 412.7 mPa·s for 10% TS (Total Solids) at 20 °C to 6.75 mPa·s for 4% TS at 55 °C [58]. Liu et al. (2012) studied rheological properties of slurry fuel prepared using municipal wastewater sludge and coal. Although the concentration was much higher than that of hot trub, the viscosity values ranged from 2750 to 1000 mPa·s [59]. There are no waste products that have similar viscosity values to those of hot trub, which makes this study an innovation. On the other hand, shear thinning is a common property.

## 4. Conclusions

1. The study allowed us to conclude the following: Hot trub is a non-Newtonian thixotropic viscous fluid characterized by maximum viscosity, equilibrium viscosity, and a hysteresis loop area.
2. The viscosity of industrial sediment was significantly lower than that of semi-technical laboratory sediment. For manufacturers of pumps used in the brewing industry, the most convenient material is that of the lowest viscosity (longer boiling precipitated sediment). For hot trub that deposits in the central zone of the tank bottom, it would be better to have higher viscosity. This rheological feature also has great advantages when composting this industrial waste. Low viscosity hot trub can be removed at lower velocities, which makes its cleaning and transport easier and cheaper.
3. Puławski hops had the highest protein and dry matter contents; therefore, it was the best to study hot trub characteristics. Mixed with water at increasing shear rate, it created a uniform suspension. Hop sediments do not contain microbial contaminants and therefore remain fully sterile.
4. The mass of precipitated hot trub depends more on the share of cereal raw materials in the batch, and less on the type of hops.
5. The shorter the boiling of wort with hops (regardless of the raw material input), the lower the share of protein and dry matter in precipitated hot trub is.
6. The use of hot water causes greater swelling of hop particles. However, hops immersed in cold water create a more stable suspension.

## Figures and Tables

**Table 1:** List of analyzed variants.

Variant Symbol	Symbol Designation
O_Przem_P	Hot trub from the industrial brewery
O_P_60	Hot trub precipitated from Pilsner malt wort and boiled for 60 min
O_L_60	Hot trub precipitated from Lager malt wort and boiled for 60 min
O_D_60	Hot trub with yeast precipitated from Pilsner malt wort and boiled for 60 min
O_P_30	Hot trub with yeast precipitated from Pilsner malt wort and boiled for 30 min
O_L_30	Hot trub precipitated from Lager malt wort and boiled for 30 min
Ch_z	Puławski hop pellets dissolved in water at 21 °C
Ch_g	Puławski hop pellets dissolved in water at 100 °C
D	Freeze-dried yeast
Ch_Pł	Puławski hop pellets
Ch_M	Magnat hop pellets
Ch_L	Lubelski hop pellets
B_Przem	Industrial wort
B_P	Pilsner malt wort
B_L	Lager malt wort
O_1	Hot trub precipitated from Pilsner malt wort and boiled 60 min
O_2	Hot trub precipitated from Lager malt wort and boiled for 60 min
O_3	Hot trub with yeast precipitated from Pilsner malt wort and boiled for 60 min
O_4	Hot trub precipitated from Pilsner malt wort and boiled for 30 min
O_5	Hot trub precipitated from Lager malt wort and boiled for 30 min
Zw	Hops dissolved in water at 21 °C
Gw	Hops dissolved in water at 100 °C

## Data Availability

Data are available from the authors.
